# Effect evaluation of case-based learning with situated cognition theory on competence training for student nurses in pediatric surgery

**DOI:** 10.1016/j.heliyon.2023.e13427

**Published:** 2023-02-02

**Authors:** Miyan Wang, Xiaohong Chen, Yuwei Yang, Haiyan Wang, Yan Yan, Xiaoying Huang, Yanli Bi, Wensha Cao, Guoxue Deng

**Affiliations:** Mianyang Central Hospital, Affiliated to School of Medicine, University of Electronic Science and Technology of China, Mianyang 621000, PR China

**Keywords:** Training effect, Reliability, Case-based learning, Situated cognition, Nursing quality, 95% CI-LL, low limit of 95% confidence interval, CBL-SCT, case-based learning with situated cognition theory, NA, not applicable

## Abstract

**Objective:**

The case-based learning with situated cognition theory (CBL-SCT) approach focuses on teaching over learning, making it suited to student nurse education. However, it is rare in student nurse training in pediatric surgery, and some subjective evaluations of the learning effect are still affected by the assessor. This study investigated the effect of the CBL-SCT approach on improving the nursing quality/safety and comprehensive performance of student nurses, and explored a method for analyzing the reliability of subjective evaluations.

**Methods:**

Thirty-six student nurses were divided into a control group and an experimental group and received seven days of orientation via conventional and CBL-SCT training, respectively. The learning effect was evaluated via examining their implementation of nursing quality criteria within the following month and their comprehensive clinical performance after six months. Among the evaluation indicators, professional skills, job competency, and professional quality were evaluated by assessors, whose scores were tested for consistency using Cronbach's alpha.

**Results:**

Among the 11 nursing quality criteria, the correct implementation of patient identification and communication (*t* = 2.257, *P* = 0.031), medication-checking (*t* = 5.444, *P* < 0.001), tumbles/bed-falling prevention (*t* = 3.609, *P* = 0.001), pressure injury prevention (*t* = 3.834, *P* = 0.001), catheter management (*t* = 3.409, *P* = 0.002), and nursing record writing (*t* = 2.911, *P* = 0.006) in the experimental group were all higher than in the control group. Six months after training, the experimental group was also higher in professional theory (*t* = 4.889, *P* < 0.001), professional skills (*t* = 2.736, *P* = 0.010), job competency (*t* = 5.166, *P* < 0.001), and professional quality (*t* = 16.809, *P* < 0.001). Cronbach's alpha test verified that the assessors' evaluations had good internal consistency and reliability for job competency (alpha = 0.847, 95% CI lower limit = 0.769), professional quality (alpha = 0.840, 95% CI lower limit = 0.759), and professional skills (alpha = 0.888, 95% CI lower limit = 0.822).

**Conclusions:**

The CBL-SCT method can help student nurses quickly change their nursing role, and Cronbach's alpha test can verify the reliability of subjective evaluations, thus indirectly reflecting the training effect equitably and objectively.

## 1 Introduction

Medical or nursing quality/safety is the primary factor that ensures patient safety [1]. The World Health Organization proposed that patient safety management is a control process that reduces unnecessary injuries related to medical treatment to the lowest risk level, which can reduce secondary injuries to patients [2,3]. Globally, important measures to address patient safety issues include the establishment of patient safety goals and standards, which are regarded as a part of the hospital accreditation process and are also needed to promote skill mastery through continuous professional training in health care [4]. For the nursing profession, it is important to train nurses at all levels in patient safety, especially for entry-level or student nurses [5].

In China, standardized training in medical professionalism for undergraduate students began in 2006, but the pilot work in standardized training for new nurses or nursing students was launched in 2016. Since then, standardized training has become an important part of nursing students’ reeducation after graduation. The standardized training for nurses is a reeducation process for undergraduate nurses, and professional training helps undergraduate nurses to transform into primary nurses as soon as possible. It is an important process to ensure that undergraduate nurses form professional ethics, develop clinical thought, and improve their practical skills. This process has become a critical factor in discipline development, and also a vital task in talent cultivation [6]. Particularly, the dearth of courses involving pediatric surgical care in nursing colleges and universities has resulted in the absence of pediatric surgical nursing ability in undergraduate nurses, so student nurses entering the pediatric surgery field need to improve their practical skills and professional ability as soon as possible to ensure independent and standardized job competency [7,8].

Standardized training can not only help nursing students master the basic theory, knowledge, and skills of clinical nursing work, but can also develop nursing students with good professional ethics, communication skills, and emergency rescue capabilities who can provide independent, standardized, and safe nursing services to patients and implement accountable holistic nursing [[9], [10], [11]], including professional care, disease observation, adjuvant treatment, psychological nursing, health education, rehabilitation direction, etc. Various problems are inevitably encountered in practical work, and only with good critical thinking ability can trainee nurses implement nursing strategies accurately. Therefore, to train qualified nurses as soon as possible, the teaching concepts and methods of standardized training are constantly innovated to ensure nursing safety.

Multiple teaching and learning strategies help to adequately equip nursing students with the necessary patient safety competencies [12,13]. Case-based learning with situated cognition theory (CBL-SCT) is a teaching method that deeply integrates case-based learning and situated cognition, namely the combination of theoretical training and clinical nursing practice, with typical true cases as the basis, progressively deeper questions as the guide, and clinical decisions as the main line of the teaching mode, so that students experience learning in real situations [14,15]. This learning mode not only enables students to experience the means and attitude to deal with events, but also enables students to strengthen their cognition of the logical reasoning process from gradually deepening questions to oriented clinical decisions. In the course of learning, students can actively find, analyze, and solve problems, which can improve their critical thinking and practical ability, and develop their ability to deal with clinical emergencies [16,17]. It has been reported that the CBL-SCT method can significantly improve the training effect [18,19], but so far there have been few reports on its use in pediatric surgical nursing training.

Learning effect evaluation is also one of the important elements of the training. Most of the evaluation indicators have clear standards and can be objectively evaluated. However, some evaluation indicators are still affected by the subjective factors of the evaluators, such as their accuracy of skill operation, job competency, level of professional quality, etc. To quickly improve the nursing quality and safety awareness of student nurses, this study investigated the effect of the CBL-SCT approach on improving the nursing quality/safety and comprehensive performance of student nurses, and further explored a method for analyzing the reliability of subjective evaluation.

## 2 Methods

### 2.1 Subjects

From Jan. 2018 to Dec. 2021 in the Department of Pediatric Surgery of Mianyang Central Hospital, affiliated to the School of Medicine, University of Electronic Science and Technology of China, we consecutively enrolled all student nurses who participated in standardized training. Initially, a total of 41 student nurses were consecutively enrolled in this investigation according to our inclusion criteria. Then, based on a random number table developed in advance, 21 were assigned to the control group and 20 to the experimental group, and they were then trained by 2 independent teaching teams. Eventually, based on the exclusion criteria, only 36 student nurses were retained for this investigation.

Inclusion criteria: All participants had completed their professional nursing education, were capable of communicating well with their patients and explaining things patiently and in easy-to-understand language, their rotation time had reached six months in the Department of Pediatric Surgery, and they agreed to participate in this study.

Exclusion criteria: The subject had a mental disorder or excessive stress, the subject terminated their internship early, the subject took sick leave of more than a month, or the subject did not participate in the theory tests and/or skill assessments in time.

### 2.2 Training implementation

A teaching team for standardized training was set up in the Department of Pediatric Surgery. The head nurse was responsible for the planning and design of routine theoretical and case-based teaching. The teaching leader was responsible for the formulation of the theoretical curriculum list, and the supervision. Senior nurses were responsible for the implementation of the theoretical curriculum, with or without the integration of clinical scenarios. All teammates had a nursing certificate with intermediate or senior professional title, bachelor's degree or above, and professional experience of more than five years. In order to implement the conventional and CBL-SCT teaching modes separately, the teaching team was divided into two independent training groups.

The training contents mainly included patient safety objectives (2019 version), the core institutions of medical quality and safety, the hospital nursing quality standards of Sichuan Province, the emergency plans of Mianyang Central hospital, the nurse training manual formulated by our hospital, etc. A total of 21 nursing quality standards have been generalized by the Nursing Quality Control Center of Sichuan Province. Our teammates implemented the courseware preparation according to its detailed training needs and standard regulations. Of them, there are 11 nursing quality standards that the Department of Pediatric Surgery focuses on, involving multiple core regulations for medication-checking, progressive patient care, shift-to-shift nursing handover, patient identification and communication, adverse events and complaint management, tumbles/bed-falling prevention, pressure injury prevention, catheter management, critical value reporting, perioperative management, and nursing record writing.

### 2.3 Training modes

Within seven days after admission, the control group was trained in the conventional mode, which was a theoretical explanation combined with a case demonstration mode via PowerPoint courseware. The training content was taught in the ward study room by a designated training group, and an in-class written test was administered. After passing all courseware examinations, these student nurses could operate in clinical practice.

At the same time, the experimental group adopted the CBL-SCT mode, which is a mode where student nurses put themselves in selected typical clinical case situations and learn relevant theoretical knowledge and practical skills through observational learning integrated with the teacher's theoretical interpretation during the entire operational process. In this teaching mode, students can deeply experience the nursing procedure and details from the clinical situation progression. Each training content was taught by another designated training group in the Hospital Clinical Skills Center, and a variety of examination methods were used, including an in-class oral test, after-class written test, and simulation examination. After passing all courseware examinations, these student nurses could operate in clinical practice. The practical and theoretical CBL-SCT program covering 11 nursing quality standards is shown in Table 1.

**Table 1:** The practical and theoretical CBL-SCT program.

Day	Teaching contents	Teaching purpose	Teaching method	Evaluating method
1st day a.m.	Overview of 10 patient safety goals and 18 core nursing systems of care	1) To master the requirements of patient safety objectives; 2) To understand the quality objectives of core nursing regulations	Lecture by PowerPoint courseware	Written test Ask occasionally
1st day p.m.	Medication-checking	To master the process of the medication-checking and use the PDA skillfully	Typical case scenario simulation	Onstage question Simulating examine
2nd day a.m.	Progressive patient care	To master the key points and evaluation criteria of graded nursing	Typical case scenario simulation	Onstage question Written test
2nd day p.m.	Shift-to-shift nursing handover	To master the specific contents and attentions of shift and succession handover	Role-playing scenario simulation	Onstage question Simulating examine
3rd day, a.m.	Patient identification and communication	To master the way to identify patients and communicate with special patients	Typical case scenario simulation	Onstage question Simulating examine
3rd day p.m.	Adverse events and complaint management	1) To understand the disposal and reporting process of Adverse events and complaint management; 2) To master the details of incident registration and preliminary handling principles	Role-playing scenario simulation	Onstage question Written test
4th day a.m.	Tumble/bed-falling prevention	To master the disposal process and preventive measures for tumble/bed-falling	Role-playing scenario simulation	Onstage question Simulating examine Written test
4th day p.m.	Pressure injury prevention	To master the disposal process and preventive measures of stress injury	Typical case scenario simulation	Onstage question Simulating examine Written test
5th day, a.m.	Catheter management	To master the management standard of the catheter and the emergency plan after the catheter removal	Role-playing scenario simulation	Onstage question Simulating examine Written test
5th day p.m.	Critical value reporting	To master the professional critical value catalog and disposal specifications	Role-playing scenario simulation	Onstage question Simulating examine Written test
6th day a.m.	Nursing record writing	To master the key points and frequency of the professional nursing records	Role-playing scenario simulation	Onstage question Written test
6th day p.m.	Perioperative management	To master the key points of preoperative and postoperative nursing, as well as the handover details of intraoperative disposition events	Role-playing scenario simulation	Onstage question Simulating examine Written test

### 2.4 Assessment of nursing quality and comprehensive performance

Within one month of completing the training, the head nurse or teaching leader evaluated the nursing quality of all student nurses according to the evaluation forms of the Nursing Quality Control Center of Sichuan Province. We divided the content of the evaluation forms into two parts: theoretical and practical. For each nursing quality standard item, the scores of the theoretical part were based on a written test, with 20 questions and a full score of 40; the scores of the practical part were based on the operational performance of the student nurses, with a full score of 60. The 11 nursing quality standard items involved 10 to 15 operational points each (see Table 2). In reference to these points, the standardized training team observed, recorded, and summarized the operational performance of the student nurse during daily operations in this month.

**Table 2:** Deduction rules for the practical part of 11 nursing quality standard items.

Nursing quality standard items	Number of Operation points	Deduction rule	Min scores	Max scores
Medication-checking	12	5 scores per point	0	60
Progressive patient care	12	5 scores per point	0	60
Shift-to-shift nursing handover	12	5 scores per point	0	60
Patient identification and communication	10	6 scores per point	0	60
Adverse events and complaint management	10	6 scores per point	0	60
Tumble/bed-falling prevention	15	4 scores per point	0	60
Pressure injury prevention	10	6 scores per point	0	60
Catheter management	12	5 scores per point	0	60
Critical value reporting	10	6 scores per point	0	60
Nursing record writing	15	4 scores per point	0	60
Perioperative management	15	4 scores per point	0	60

After a six-month rotational period, the standardized training team evaluated the comprehensive performance of all student nurses according to the Evaluation Table of Clinical Comprehensive Performance formulated by the Nursing Department of Mianyang Central Hospital. Comprehensive performance was assessed from four aspects: professional theory, professional skills, job competency, and professional quality [20]. Professional theory was scored according to their examination papers. The professional skills were scored by three assessors based on the on-site operations of the student nurses in accordance with established assessment procedures. The job competency of student nurses was evaluated by five assessors based on the five aspects of job competence, cooperation/innovation, professional knowledge/skills, initiative/responsibility, and diligent attendance, with four grades in each aspect and five scores in each grade. The professional quality assessment was conducted by five assessors using a five-point Likert-type scale based on the three aspects of professional ethics, clinical manifestations, and communication ability [21]. The five assessors had a consistent understanding of the assessment rules and reached a consensus to ensure the homogeneity of the scoring.

### 2.5 Reliability verification of subjective scores from the assessors

Cronbach's alpha test was also used to verify the reliability of the three evaluation indexes involving subjective factors (i.e., professional skills, job competency, and professional quality), so as to evaluate the credibility of the scoring results. The reliability of the scoring results from three to five assessors was presented as alpha (95% confidence lower limit [95% CI-LL]). When the alpha was ≤0.700, 0.701–0.800, 0.801–0.900, or >0.900, it indicated no, slight, fair, or very good consistency and reliability, respectively. At the same time, we also observed the change in the reliability value after dropping a designated assessor, which could reflect the contribution of the assessor to the reliability value.

### 2.6 Statistical analysis

SPSS22.0 and MedCalc18.2 software were used for the statistical analysis. The categorical data were expressed as frequency (%), and the difference between the two groups was tested using a four-fold table χ^2^ test. The measurement data were confirmed to be normally distributed, expressed as mean plus/minus standard deviation, and the difference between the two groups was tested with an independent sample *t*-test. Cronbach's alpha test was used for three to five assessors to verify the consistency and reliability of the scoring results and was presented as alpha (95% CI-LL). P < 0.05 indicated that the difference was statistically significant.

### 2.7 Ethical review

This study included experimentation on human subjects, which conducted in the Department of Pediatric Surgery, Mianyang Central Hospital, School of Medicine, University of Electronic Science and Technology of China. The study protocol was approved by the Ethics Committee of Mianyang Central Hospital in accordance with the Helsinki declaration of the World Medical Association (approval No. S2018085). Informed consent for administration of an authentic scenarios or simulation scenarios was obtained from the parents of infants enrolled in the study.

## 3 Results

### 3.1 Basic information on the two groups of standardized training student nurses

A total of 36 standardized training student nurses were randomly divided into 2 groups (shown in Table 3). There were no significant differences in age (*t* = 1.724, *P* = 0.094), sex (χ^2^ = 0.144, *P* = 0.704), or academic career (χ^2^ = 0.538, *P* = 0.463) between the two groups.

**Table 3:** Comparison of sex, age, and education background between the two groups.

Observed indices	Control group (n = 18)	Experimental group (n = 18)	χ^2^/t
Age (years)	21.3 ± 1.1	22.3 ± 2.3	1.724, 0.094
Male/Female [n (%)]	4(22.2)/14(77.8)	5(27.8)/13(72.2)	0.144, 0.704
College/Bachelor [n (%)]	14(77.8)/4(22.2)	12(66.7)/6(33.3)	0.538, 0.463

Notes: Independent sample *t*-test was used for age, and χ2 test was used for the else. There were no statistical differences in these observation items between the Control group and the Experimental group.

### 3.2 Training effect difference of nursing quality standards between the two groups

**Fig. 1:**
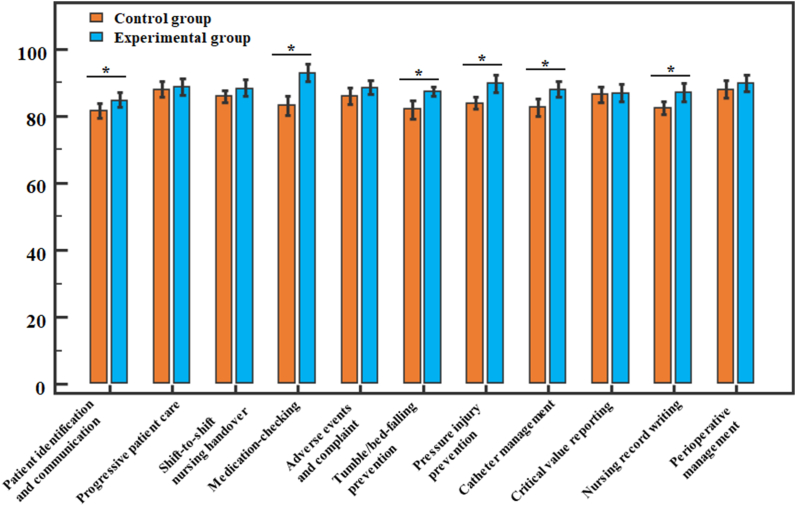
The score differences in 11 nursing quality standards between the 2 groups. Notes: *P < 0.05. The scores of the experimental group for 6 of 11 nursing quality standards were higher than the control group, indicating that the teaching mode of CBL-SCT could promote student nurses' mastery of nursing quality standards.

The first evaluation of the implementation of student nurses' quality standards was conducted within the first month of training completion. Among the 11 nursing quality standards that the department focused on, the experimental group scored higher than the control group on 6 (shown in Fig. 1): patient identification and communication (*t* = 2.257, *P* = 0.031), medication-checking (*t* = 5.444, *P* < 0.001), tumbles/bed-falling prevention (*t* = 3.609, *P* = 0.001), pressure injury prevention (*t* = 3.834, *P* = 0.001), catheter management (*t* = 3.409, *P* = 0.002), and nursing record writing (*t* = 2.911, *P* = 0.006). There was no significant difference in the scores for other nursing quality standards between the two groups (*t* = 0.244–1.713, *P* = 0.096–0.809). This showed that the teaching mode used with the experimental group could promote student nurses’ mastery of nursing quality standards to a certain extent.

### 3.3 Evaluation difference of clinical comprehensive manifestations between the two groups

**Fig. 2:**
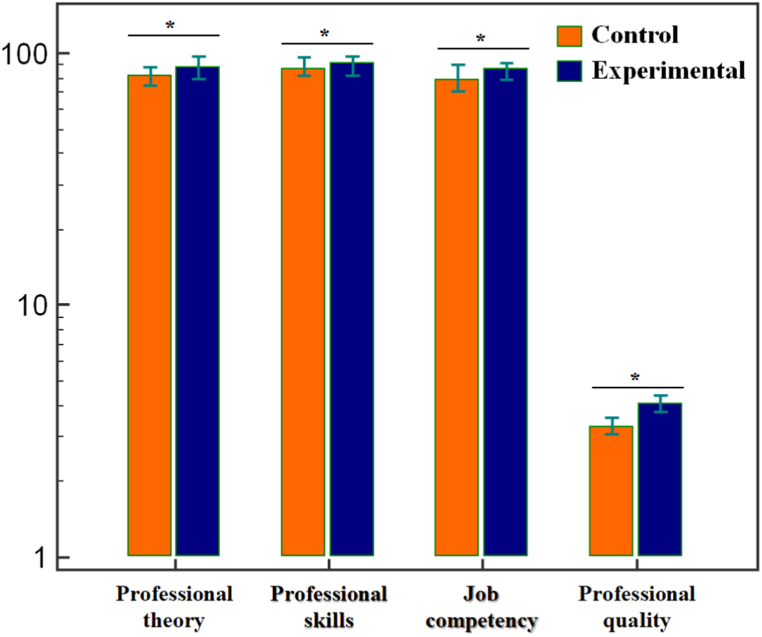
The score differences in clinical comprehensive manifestations between the two groups. Notes: *P < 0.05. The scores of all four aspects in the experimental group were higher than the control group, suggesting that the teaching mode of CBL-SCT was helpful for the long-term cultivation of comprehensive ability.

After completing the six-month rotation, we evaluated the clinical comprehensive manifestations of the student nurses. The results showed (Fig. 2) that the experimental group was higher than the control group in all aspects of clinical comprehensive manifestations: professional theory (*t* = 4.889, *P* < 0.001), professional skills (*t* = 2.736, *P* = 0.010), job competency (*t* = 5.166, *P* < 0.001), and professional quality (*t* = 16.809, *P* < 0.001). This suggested that the teaching mode used with the experimental group could also promote the long-term cultivation of student nurses’ comprehensive ability.

### 3.4 Reliability verification of subjective scores from the assessors to all student nurses

According to Cronbach's alpha test (Table 4), the reliability coefficient of professional skills was alpha = 0.888 (95% CI-LL = 0.822), that of job competency was alpha = 0.847 (95% CI-LL = 0.769), and that of professional quality was alpha = 0.840 (95% CI-LL = 0.759), both with fair consistency and reliability. This suggested that Cronbach's alpha test might be a suitable analysis method for determining the reliability of subjective evaluation.

**Table 4:** Consistent reliability of the student nurses' clinical comprehensive performance scores.

Observed variables	professional skills		Job competency		Professional quality	
	Alpha	Change	Alpha	Change	Alpha	Change
Consistency reliability	0.888	NA	0.847	NA	0.840	NA
95% lower confidence limit	0.822	NA	0.769	NA	0.759	NA
Effect of dropping Assessors						
Assessor 1 dropped	0.770	−0.118	0.794	−0.054	0.811	−0.029
Assessor 2 dropped	0.858	−0.030	0.811	−0.037	0.857	0.017
Assessor 3 dropped	0.889	0.001	0.859	0.013	0.796	−0.044
Assessor 4 dropped	NA	NA	0.799	−0.048	0.798	−0.042
Assessor 5 dropped	NA	NA	0.809	−0.037	0.765	−0.075

Note: “Alpha” is the statistic value of Cronbach's alpha test, and its numerical magnitude is used to directly assess the consistent reliability of the student nurses' scores. When alpha is high than 0.700 or 0.800, the consistent reliability reaches a slight or fair degree. “Change” looks at the change in credibility after dropping this assessor's scores, and can reflect the contribution of the assessor to the Alpha coefficient. It was found that the assessors rating scores for student nurses in job competency, professional quality and professional skills reached a fair degree of reliability.

## 4 Discussion

This investigation demonstrated that the training mode of CBL-SCT is not only beneficial to student nurses working to master the norms of the nursing quality standards, but can also help student nurses to improve their professional theory, professional skills, job competency, and professional quality. The most prominent characteristic of this investigation was its adoption of the Cronbach's alpha test to evaluate the homogeneity of student nurses' scores obtained from different assessors. Technically speaking, Cronbach's alpha is a reliability coefficient of consistency or homogeneity but is not a statistical test [22,23]. Just like psychological, educational, or other medical surveys, the scoring results of student nurses are also affected by the subjectivity of the assessors [[24], [25], [26]], so validation of the consistency or homogeneity of the assessors is particularly important. We used Cronbach's alpha to test the consistency and reliability of the scores from different assessors to verify the homogeneity of the evaluation of training effects. The results of this investigation showed that the evaluation homogeneity of the training effect was better reflected, at least in the evaluation of the student nurses' professional skills, job competency, and professional quality.

All of the above achievements seem to be attributable to the CBL-SCT mode we adopted. This is a teaching mode based on intensive training with real cases [27,28]. We utilized concepts from case-based learning and situated cognition theory to improve student nurses' professional education and personal growth through inquiry, reflection, and shared discovery during the implementation of typical cases. However, how to solve the problem of homogenous teaching of enrolled student nurses at different times is key to ensuring the reliability of the findings. To this end, we carried out the following three measures for teaching quality control: 1) established a unified goal for all layers of teaching content and process, 2) used completely authentic case scenarios or simulated scenarios, and 3) selected typical cases. The teaching content was divided into three layers: structure, process, and result. The teaching process was also divided into three layers: perception, cognition, and application [29]. We established a unified goal at all layers, which was requiring student nurses to be familiar with the structural layer (perception), master the process layer (cognition), and clearly evaluate the result layer (application). The cases were based on real patients and their families, doctors, and nurses in the hospital, and the teaching was carried out from real or simulated scenarios, in order to train student nurses in teamwork and communication skills in real situations [30,31]. The selection of situational cases was adopted in three ways: the first way was to quote typical cases for analysis and discussion, and summarize the theoretical points that needed to be mastered [32]; the second way was to enter the real scene of the ward to observe and emulate the actual operation, and then combine it with theory to find problems and summarize the essential points and difficulties; the third way was to design a full simulation [33], contrapose the adverse events and complaints, and let student nurses determine the correct response in a tense atmosphere. In addition, in the process of case-based learning, it was necessary to design simulated scenarios with various changes to cultivate student nurses’ ability to deal with emergent events.

The CBL-SCT mode could promote the rapid transformation of the role of training nurses. Our investigation showed that CBL-SCT courses combined with the practical characteristics of the profession played an obvious role in improving the training effect, leading to the student nurses in the experimental group significantly improving their professional skills, specialty theory, post competence, and professional quality. The enhancement of the training effect was more beneficial to the rapid improvement of the independent practice ability of student nurses [34]. Therefore, the CBL-SCT mode can help student nurses to grasp the nursing quality standards firmly at the initial stage of entering the pediatric surgery department, and then to complete their clinical nursing work in practice calmly, orderly, and efficiently.

The CBL-SCT mode can also enhance students' sense of real experience and stimulate their interest in learning. With the improvement of nursing education, nurses are not limited to clinical practice but also need to assist doctors to promote the rapid recovery of patients. Introducing student nurses to real situational experiences is more conducive to actively guiding their ability to discover, analyze, and solve problems independently [35,36], as well as to facilitating nurse clinicians' transition to the nurse educator role [37]. Studies have shown that adverse events are closely related to patient safety and care quality, but about 50% of them can be prevented [38,39]. Because student nurses had poor clinical practice ability, weak critical thinking ability, a lack of communication skills, and an absence of teamwork awareness, they had become the “main force” leading to the occurrence of safety risks [[40], [41], [42]]. Our results showed that through the teaching-learning mode of CBL-SCT, student nurses in the experimental group significantly improved their practice skills in six nursing quality standards. This proved that CBL-SCT could help student nurses to master clinical skills and the processing ability, and has the advantage of guaranteeing nursing quality and safety. Therefore, the CBL-SCT mode enables student nurses to experience patients’ feelings, nursing processes, and prominent problems in real scenes, and intuitively understand the importance of patient safety risk management, especially the risk management of tumbles/bed-falling, pressure injuries, catheter shedding, etc.

Strengths of this study: The study focused on student nurses' training in pediatric surgery. Because of the particularity of the pediatric group—for example, their low cognitive ability, lack of expression, and poor compliance—the CBL-SCT mode can be more intuitive, specific, and flexible in showing the characteristics of pediatric nursing, highlighting its particularity, and shifting the focus of training from teaching to learning. This makes it especially suitable for pediatric nursing training. In addition, we successfully applied Cronbach's alpha test to evaluate the consistency and objectivity of the evaluators' subjective scores, providing a reference method for researchers to expand more applicable tools for the assessment of fairness.

Limitations of this study: The study focused on the difference between the conventional and CBL-SCT teaching modes, without considering the impact of differences in the basic quality of student nurses' and teachers’ ability on the results. In addition, due to the limited number of student nurses enrolled in the department, the research conclusions need to be further verified with large samples.

## 5 Conclusions

The World Health Organization advocates that health professionals should have the competency to guarantee patient safety, and take this as the basis for carrying out patient safety education and estimating the job competency of health professionals. Our study showed that the CBL-SCT mode played a significant role in nursing education, helping student nurses master nursing quality standards quickly and improve their competence in daily work. Moreover, using Cronbach's alpha to test the reliability of evaluations can better reflect the homogeneity and objectivity of assessors' evaluations of students.

## Author contributions statement

Conceptualization: Miyan Wang, Yuwei Yang, and Haiyan Wang.

Data curation: Xiaohong Chen, Yan Yan, Xiaoying Huang, and Yanli Bi.

Formal analysis: Miyan Wang and Yuwei Yang.

Funding acquisition: Miyan Wang and Yuwei Yang.

Investigation: Wensha Cao and Guoxue Deng.

Writing - original draft: Miyan Wang.

Writing - review & editing: Yuwei Yang and Haiyan Wang.

## Funding statement

This research was financially supported by a project of Mianyang Central Hospital, affiliated to 10.13039/100008235School of Medicine, 10.13039/501100005408University of Electronic Science and Technology of China (2021YJ005), and the Science & Technology Department of Sichuan Province, China (2019YJ0701). The authors have no other relevant affiliations or financial involvement with any organization or entity with a financial interest in or financial conflict with the subject matter or materials discussed in the manuscript apart from those disclosed.

## Data availability statement

The datasets used and/or analyzed during the current study are available from the corresponding authors on reasonable request.

## Declaration of competing interest

The authors declare that they have no known competing financial interests or personal relationships that could have influenced the work reported in this paper.
